# Tonic suppression of *PCAT29* by the IL-6 signaling pathway in prostate cancer: Reversal by resveratrol

**DOI:** 10.1371/journal.pone.0177198

**Published:** 2017-05-03

**Authors:** Raheem F. H. Al Aameri, Sandeep Sheth, Entkhab M. A. Alanisi, Vikrant Borse, Debashree Mukherjea, Leonard P. Rybak, Vickram Ramkumar

**Affiliations:** 1 Department of Pharmacology, Southern Illinois University School of Medicine, Springfield, Illinois, United States of America; 2 Department of Medical Microbiology, Immunology, and Cell Biology, Southern Illinois University School of Medicine, Springfield, Illinois, United States of America; 3 Department of Surgery, Southern Illinois University School of Medicine, Springfield, Illinois, United States of America; University of South Alabama Mitchell Cancer Institute, UNITED STATES

## Abstract

Prostate cancer (PCa) is the second leading cause of cancer deaths in men. A better understanding of the molecular basis of prostate cancer proliferation and metastasis should enable development of more effective treatments. In this study we focused on the lncRNA, prostate cancer associated transcript 29 (*PCAT29*), a putative tumor suppressive gene. Our data show that the expression of *PCAT29* was reduced in prostate cancer tumors compared to paired perinormal prostate tissues. We also observed substantially lower levels of *PCAT29* in DU145 and LNCaP cells compared to normal prostate (RWPE-1) cells. IL-6, a cytokine which is elevated in prostate tumors, reduced the expression of *PCAT29* in both DU145 and LNCaP cells by activating signal transducer and activator of transcription 3 (STAT3). One downstream target of STAT3 is microRNA *(miR)-21*, inhibition of which enhanced basal *PCAT29* expression. In addition, we show that resveratrol is a potent stimulator of *PCAT29* expression under basal condition and reversed the down regulation of this lncRNA by IL-6. Furthermore, we show that knock down of *PCAT29* expression by siRNA in DU145 and LNCaP cells increased cell viability while increasing *PCAT29* expression with resveratrol decreased cell viability. Immunohistochemistry studies showed increased levels of STAT3 and IL-6, but low levels of programmed cell death protein 4 (PDCD4), in prostate tumor epithelial cells compared to adjacent perinormal prostate epithelial cells. These data show that the IL-6/STAT3/*miR-21* pathway mediates tonic suppression of *PCAT29* expression and function. Inhibition of this signaling pathway by resveratrol induces *PCAT29* expression and tumor suppressor function.

## Introduction

About 2% human genome sequences are functional genes -which coding for functional proteins- and regulatory elements, while other of the human genome sequence is considered non-coding sequence with different functions. [1] Recently, multiple studies have revealed new forms of RNA widely known as long non-coding RNA (ncRNA) transcribed from non-coding sequence in DNA [2]. These ncRNAs have different functions and can be divided according to their sizes into two groups. These include short ncRNA (less than 200 nucleotides in length), such as microRNA (miRNA) and interference RNA (siRNA) and long ncRNA (more than 200 nucleotides) such as long non-coding RNA (lncRNA) [3]. There are also other classes of RNA which have housekeeping function in protein synthesis, such as transfer RNA (tRNA) and ribosomal RNA (rRNA) [4–6]. The functions of lncRNA are still largely unknown but some studies have linked them to different cellular roles including epigenetics regulation of transcription, such as *ANRIL* and *HOTAIR*, which interact with PRC1 and PRC2 transcription complex and repress gene transcription [7,8]. Enhancer-derived RNA (eRNA) acts as a transcription enhancer which are important role in transcription activation in androgen positive prostate cancer cells [9]. In addition, *MEG3* is a tumor suppresser whose activity is dependent on the p53 protein [10–12]. *MALAT* and *NEAT* lncRNAs are implicated in mRNA splicing, editing and exporting mRNA to cytoplasm [13,14]. In addition, the oncogene *PCAT1* activates cell proliferation and inhibits BRCA2 [8].

Prostate cancer (PCa) is the second leading cause of cancer deaths in in men [15,16]. Several risk for developing PCa include genetic modifications in oncogenes such as STAT3 [16], modification in tumor suppressor genes such as phosphatase and tensin homolog deleted on chromosome 10 (PTEN) gene, or mutations in androgen receptors [17–19]. Epigenetic alterations, including DNA methylation in tumor suppresser genes promoters and modification in histone modifying enzymes also contribute to PCa carcinogenesis [20]. Recent studies have showed that alteration in different lncRNAs, such as *PTENP1* [21,22], *Linc00963* [23], *PCGIM1* [24], *PRNCR1* [25], *CBR-3AS1* [26], *CTP1 AS* [27], *GAS5* [28], *ANRIL* [29], *ANRASSF1* [30] and *PCAT1* [8, 31] are associated with PCa. LncRNAs, such as prostate cancer associated transcript (*PCAT1* and *PCAT3)*, increase cancer cells proliferation [8,31]. However, another LncRNA, *PCAT29* exhibits tumor suppressor features in prostate cancers by decreasing the proliferation and migration of PCa [32]. Studies have reported that both LncRNA and microRNA, such as *PCAT1* and *miR-3667*, can act synergistically to regulate PCa progression [1]. Furthermore, lncRNA and microRNA activate RNA degradation machinery and recruit STAU1 protein to enable RNA degradation [33]. While many studies have implicated *PCAT* lncRNAs in prostate cancer [31], their exact roles in the development of PCa remain unclear.

This study focuses on the regulation of *PCAT29*, a tumor suppressor lncRNA by IL-6 in prostate cancer cells. We show that IL-6 reduced *PCAT29* mRNA by activating STAT3 and *miR-21*. Co-regulation of STAT3 and *miR-21* was observed in human prostate samples. Furthermore, the chemopreventative agent, resveratrol, blocked IL-6 reduction of *PCAT29*, by interfering with the STAT3 and *miR-21* signaling.

## Materials and methods

### Ethics statement

All studies involving clinical specimen were conducted in accordance with federal regulations and using a protocol authorized by The Tissue Banking Facility of Southern Illinois University School of Medicine and approved by The Springfield Committee for Research Involving Human Subjects (SCRIHS).

### Materials

Resveratrol was purchased from Sigma-Aldrich (R5010) whereas, IL-6 was bought from Life Technologies (10395HNAE). Antibodies: PDCD4 rabbit monoclonal antibody (cat# 9535) and pSTAT3 mouse monoclonal antibody (cat# 4113) were purchased from Cell Signaling Technology^®^, while STAT3 mouse monoclonal (cat# sc-8019) and β-actin mouse monoclonal (cat# sc-69879) antibodies were obtained from Santa Cruz Biotechnology. All antibodies for Western blotting were used in the dilution of 1:1000 except β-actin, which was used in the dilution of 1:10,000. IL-6 mouse monoclonal antibody was purchased from Novus Biologicals (cat# nbp1-47810) and used in the dilution of 1:50 for immunohistochemistry. Supplies for cell culture: RPMI 1640 media and complete keratinocyte serum-free media (K-SFM) were from Gibco, penicillin/streptomycin were obtained from ThermoFisher Scientific, while fetal bovine serum (FBS) was from Atlanta Biologicals. Synthetic anti-*miR-21* oligonucleotide, STAT3 siRNA and their negative control were purchased from Ambion, while *PCAT29* siRNA and their negative control were purchased from Dharmacon^™^. The TRI reagent for RNA isolation was purchased from Sigma-Aldrich. The items for real-time RT-PCR such as iScript cDNA Synthesis Kit was purchased from Bio-Rad, while Fast SYBR^™^ Green Master Mix, TaqMan^®^ MicroRNA Reverse Transcription Kit and TaqMan^®^ Universal PCR Master Mix were purchased from and Applied Biosystems. CellTiter 96^®^ AQueous One Solution Cell Proliferation Kit for determining cell viability was purchased from Promega. Immunohistochemistry was performed using ImmunoCruz^™^ ABC Staining System which was purchased from Santa Cruz Biotechnology.

### Cell culture

Androgen-insensitive human prostate carcinoma DU145 cells, androgen-sensitive human prostate carcinoma LNCaP cells and immortalized human prostate epithelial RWPE-1 cells, were kindly provided by Dr. Daotai Nie (SIU School of Medicine, Springfield, IL). DU145 and LNCaP cells were cultured in RPMI 1640 media (Gibco) supplemented with 10% fetal bovine serum (Atlanta Biologicals), 50 units/ml penicillin and 50 μg/ml streptomycin (ThermoFisher Scientific). RWPE-1 cells were cultured in complete K-SFM which contains 50 μg/ml of bovine pituitary extract (BPE) and 5 ng/ml epidermal growth factor (EGF) (Gibco), plus 50 units/ml penicillin and 50 μg/ml streptomycin. All cell lines were grown at 37°C in the presence of 5%CO_2_ and 95% ambient air. All experiments were performed on sub-confluent monolayers.

### Cell viability assay (MTS Assay)

Cell viability of DU145 and LNCaP cells was assessed using CellTiter 96^®^ AQueous One Solution Cell Proliferation Assay kit (Promega), as per manufacturer’s protocol. Briefly, 2,500 cells per well were seeded in a 96-well plate. Twenty four hours after they were seeded, the cells were treated and allowed to grow for another 24 h, after which 20 μl of CellTiter 96^®^ AQueous One Solution reagent was added to each well containing cells with100 μl media. Cell were then incubated for 2 to 3 h and absorbance was measured at 490 nm using an ELISA plate reader. The production of the colored formazan product (absorbance) is directly proportional to the number of viable cells in culture and is expressed as percent cell viability relative to control.

### Western blot analysis

Protein expression was determined using Western blotting technique as previously described [34]. Briefly, cells were seeded in 6-well plates and incubated until 70–80% confluence. At the end of the treatment, cells were washed once with ice-cold 1X PBS and whole-cell lysates were prepared by homogenizing in ice-cold lysis buffer (150 mM NaCl, 50 mM Tris-HCl, 1.0% NP-40, 0.5% sodium deoxycholate, 0.1% sodium dodecyl sulfate) containing protease inhibitor cocktail (Sigma-Aldrich) and phosphatase inhibitor 2 and 3 (Sigma-Aldrich). Protein concentration was determined by Bradford method and equal amount of protein was resolved by SDS polyacrylamide gel electrophoresis. The proteins were then transferred on to nitrocellulose membrane and probed with specific primary antibody. Blots were then incubated with species-specific fluorescent-tagged IgG secondary antibody and scanned and visualized using LI-COR Odyssey^®^ imaging system (LI-COR Biosciences). Each band was normalized to the corresponding total STAT3 or β-actin bands. Densitometric analysis of the bands was performed using ImageJ software and results were plotted as percent of control where control was considered as 100%.

### Oligonucleotide and short interfering (si) RNA transfection

Synthetic *anti-miR-21* (30 nM) (ID# AM17000, Ambion), STAT3 siRNA (10 nM) (ID# AM16708, Ambion), *PCAT29* siRNA (30 nM) (ID# SHEKJ-000001, Dharmacon^™^) and their negative controls (scramble) were transferred into the cells using Lipofactamine^®^ RNAiMAX transfection reagent (Life Technologies^™^), according to manufacturer’s protocol. Briefly, cells were seeded in a 6-well plate and incubated until they were 60–70% confluence. When the cells were ready, anti-*miR-21*, STAT3 siRNA, *PCAT29* siRNA and their respective negative controls were diluted in 150 μl of serum free media and incubated with 9 μl of Lipofactamine^®^ RNAiMAX for 5 min to permit the formation of transfection complex. The transfection complex was then added to the cells which were then incubated for 24 h after which they were treated with IL-6 and resveratrol for indicated time. Cell were then collected for Western blotting or real-time PCR studies to detect various proteins or RNAs, respectively.

### RNA isolation

Total RNA was extracted from the cells using 500 μl of TRI reagent (Sigma). Chloroform (100 μl) was added to the TRI reagent and the tube was shaken vigorously for 15 seconds and centrifuged at 12,000 rpm for 15 min. Top aqueous layer, which contains RNA, was extracted to which 0.5 ml ice-cold isopropanol was added and the samples were centrifuged at 12,000 rpm for 10 min. Isopropanol was carefully removed and the pellet was washed with 75% ethanol in DEPC-treated H_2_O. The samples where then centrifuged again at 12,000 rpm for 10 min. The ethanol was removed and the tube was air dried briefly. The RNA pellet was resuspended in nuclease free water and RNA levels were determined based on their optical density using a Nanodrop^®^ ND-1000 Spectrophotometer.

### Real-time RT-PCR

Total RNA (1 μg) was converted to cDNA using iScript cDNA Synthesis Kit (Bio-Rad). The reaction mixture was set up as follows: 1 μg of total RNA, 4 μl of iScript reaction mix, 1 μl of iScript reverse transcriptase, nuclease free water to bring the total volume to 20μl. Reverse transcription was performed at 25°C for 5 min (priming), then 42°C for 30 min (revers transcription reaction) followed by 85°C for 5 min (reverse transcription reaction inactivation). This cDNA reaction mix was used for real-time PCR StepOnePlus^™^ Real-Time PCR Systems (Applied Biosystems). Each reaction mixture contained 0.5 μl of cDNA, 5 μl of Fast SYBR^™^ Green Master Mix (Applied Biosystems), 0.3 μl from each forward and reverse primers and 3.9 μl nuclease-free water to make up the volume to 10 μl. The reactions were incubated at 95°C for 20 sec (hold), followed by 40 cycles of 95°C for 3 sec (denature) and 60°C for 30 sec (anneal/extend). Specific primer pairs for *PCAT29* were used for the reactions and mRNA expression levels were normalized to the levels of *GAPDH*. The primer sets were purchased from Invitrogen and were as follows:

*H*. *sapiens*-*PCAT29* (forward): 5’-TCTGCTGAGACCCAGTGC-3’

       (reverse): 5’-TTCTCTCACATTTCATTCACC-3’,

*H*. *sapiens*-*GAPDH* (forward): 5’-AATCCCATCACCATCTTCCA-3’

       (reverse): 5- TGGACTCCACGACGTACTCA-3’

The cycle threshold (Ct) values were used to analyze the results of real-time PCR. *PCAT29* expression was calculated using the 2^-ddCt^ method relative to *GAPDH* and results were reported as fold change.

### TaqMan^®^ real-Time PCR

Total RNA was isolated from the cells using a protocol previously described [35]. Total RNA (100 ng) was reverse transcribed (RT) to generate *miR-21* cDNA using the TaqMan^®^ MicroRNA Reverse Transcription Kit (Applied Biosystems). Each RT reaction contained 1 μl of 1X RT specific primer for *miR-21* and *U6*, 1.5 μL 1X RT reaction buffer, 0.15 μl of 100 mM dNTPs, 50 U/μl MultiScribe reverse transcriptase, 3.8 U/μl RNase inhibitor, 100 ng RNA and RNase-free water to make the final volume of 15 μl. The reaction mix was then incubated for 30 min at 16°C, 30 min at 42°C and 5 min at 85°C. The real-time PCR was performed using Applied Biosystem StepOnePlus^™^ real-time system using TaqMan^®^ PCR kit (Applied Biosystem). After RT step, 1 μl of cDNA was combined with 0.6 μl of 20X TaqMan^®^ primers (forward and reverse) and 5 μl TaqMan^®^ Universal PCR Master Mix in 10 μl final volume. The amplification was performed by incubation at 95°C for 10 min followed by 40 cycle of 95°C for 15 sec and 60°C for 1 min. The 2^-ddCt^ was used to calculate the *miR-21* expression and *U6* snRNA was used as internal control.

### Immunohistochemistry

Formalin-fixed paraffin embedded (FFPE) human prostate tumor and their paired perinormal sections on glass slides were obtained from The Tissue Banking Facility of Simmons Cancer Institute of SIU School of Medicine. Immunohistochemistry was performed using ImmunoCruz^™^ ABC Staining System (Santa Cruz Biotechnology) for the detection of PDCD4, pSTAT3 and IL-6. Antigen retrieval was performed by first deparaffinizing and rehydrating the tissue sections and then immersing them in 10 mM sodium citrate buffer (pH 6) for 10 min in a 90°C water bath. After cooling down to room temperature, the tissue sections were incubated with 0.1% H_2_O_2_ for 10 min at room temperature to block the endogenous peroxidase activity. The sections were then washed twice with 1X PBS and blocked with 1.5% goat serum diluted in 1X PBS for 1 h at room temperature. The tissue sections were then incubated with 100 μl of respective primary antibodies (dilutions: 1:100 for PDCD4, 1:50 for pSTAT3 and 1:50 IL-6) overnight at 4°C. Next day, the sections were washed twice with 1X PBS and incubated with biotinylated secondary antibody for 30 min at room temperature. To identify the peroxidase activity, the section were first incubated with avidin and biotinylated horseradish peroxidase enzyme (AB reagent) for 10 min and then with peroxidase substrate until desired stain intensity develops. The sections were then washed in deionized water, counterstained with hematoxylin, dehydrated with ethanol and mounted using permanent mounting media. The tissue sections were imaged using an Olympus light microscope (Olympus imaging America Inc.) using Olympus DP controller software.

### Statistical analysis

Statistical analysis were performed either using analysis of variance (ANOVA) followed by Bonferroni *post hoc* correction for multiple comparisons or by paired Student’s t-test using GraphPad Prism software 6.0. The value of *p*<0.05 was considered as statistically significant. The error bars shown in the figures represent standard error of mean (SEM).

## Results

### Down-regulation of *PCAT29* expression in prostate cells and prostate tissues

**Fig 1:**
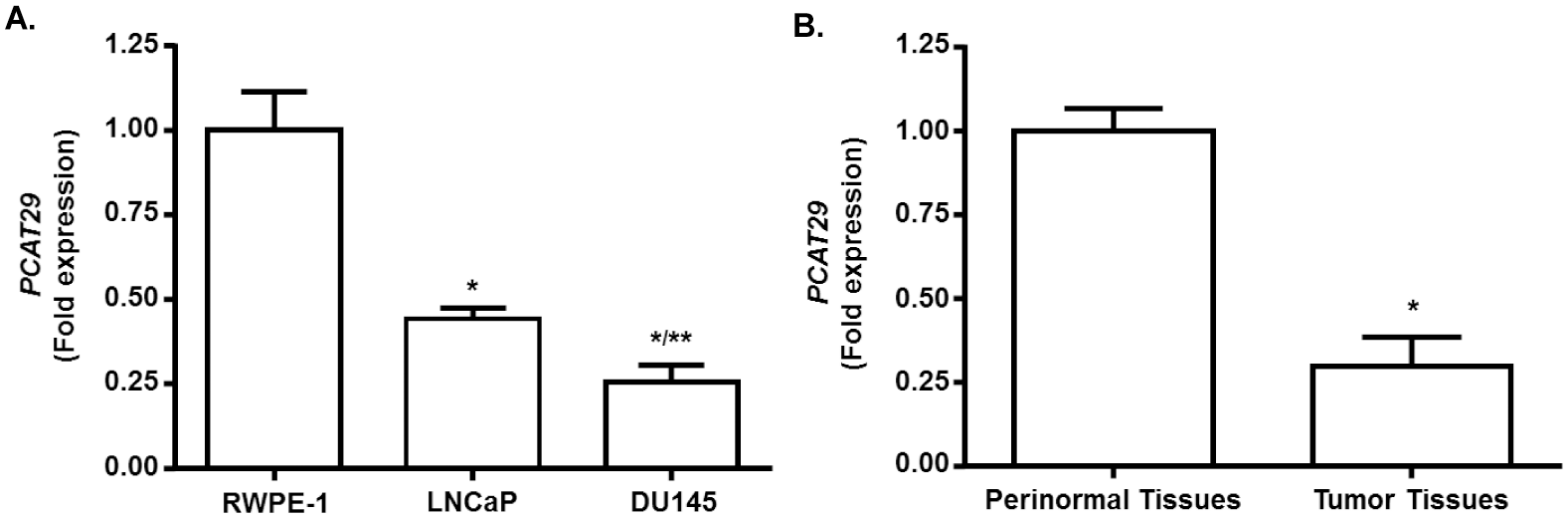
Reduced expression of *PCAT29* expression in prostate cancer cells and tumor tissues. **(A)** Relative mRNA levels of *PCAT29* in normal human prostate epithelial cells (RWPE-1) and prostate cancer cells (LNCaP and DU145). The levels of *PCAT29* were significantly reduced in prostate cancers cells compared to RWPE-1 cells. (n≥3) **(B)** Significant reductions in *PCAT29* levels in normal prostate tissues compared to prostate cancer. Data are presented as the mean ± SEM of 4 prostate samples. Asterisks (*) indicate statistically significantly difference (p < 0.05) from RWPE-1 and from normal prostate tissue, respectively. (**) indicate statistically significantly difference (p < 0.05) from LNCaP cells.

Previous studies have reported that *PCAT29* was expressed at low levels in DU145 and LNCaP cells [36]. To evaluate the level of *PCAT29* expression, we examined its expression in DU145, LNCaP prostate cancer cells and normal prostate cells, RWPE-1. The results showed that *PCAT29* expression, normalized to that in RWPE-1 cells, were 44.0 ± 3.1% and 25.6 ± 1.8%, respectively, in LNCaP and DU145 cells, cultured under similar conditions. These results show reduced expression of *PCAT29* expression in prostate cancer cells than normal prostate cells. Furthermore, the statistically significant difference in levels of *PCAT29* in the more aggressive DU145 cell line, compared to LNCaP cells, implicates down-regulation of *PCAT29* in oncogenesis (Fig 1A).

We next examined the expression of *PCAT29* in human prostate cancer tissues. These prostate tissue specimens were obtained from Tissue Banking Facility of Simmons Cancer Institute of SIU School of Medicine (Springfield, IL, USA). Fig 1B shows that *PCAT29* expression was higher in normal than in tumor tissues. The relative expression was 0.29 ± 0.15 in tumor tissues as compared to normal tissues.

### IL-6 down-regulates *PCAT29* expression on prostate cancer cells

**Fig 2:**
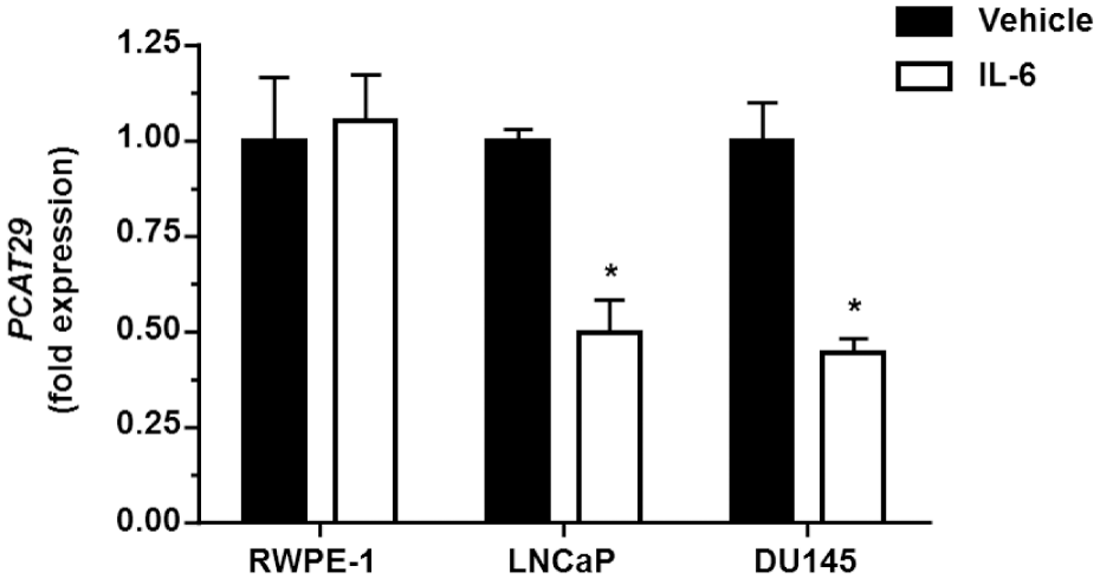
IL-6 reduced the expression of *PCAT29* in prostate cancer but not normal cells. DU145 and LNCaP cells were treated with IL-6 (10 ng/ml) for 24 h, following which the expression of *PCAT29* was determined by real-time RT-PCR. Both DU145 and LNCaP cells showed decreased *PCAT29* expression after IL-6 treatment. There was no difference in *PCAT29* expression following IL-6 treatment in RWPE-1 cells. Data are presented as the mean ± SEM of at least 3 independent samples. Asterisks (*) indicates statistically significantly difference (p < 0.05) from vehicle groups.

IL-6 plays a crucial role in the differentiation of human prostate carcinoma and benign prostatic hyperplasia. [37]. To evaluate the effect of IL-6 on *PCAT29* activity, prostate DU145, LNCaP and normal prostate cell RWPE-1 cells were treated with IL-6 (10 ng/ml) for 24 h and *PCAT29* expression was determined by qPCR. The results showed significant reductions in *PCAT29* expression for both DU145 and LNCaP cells following IL-6 treatment for 24 h. Similar treatment of RWPE-1 cells with IL-6 did not alter *PCAT29* expression. The relative expression of *PCAT29* induced by IL-6 was 44.4 ± 4.0%, 47.9 ± 7.9% and 100% in DU145, LNCaP and RWPE-1 cells, respectively (Fig 2).

**Fig 3:**
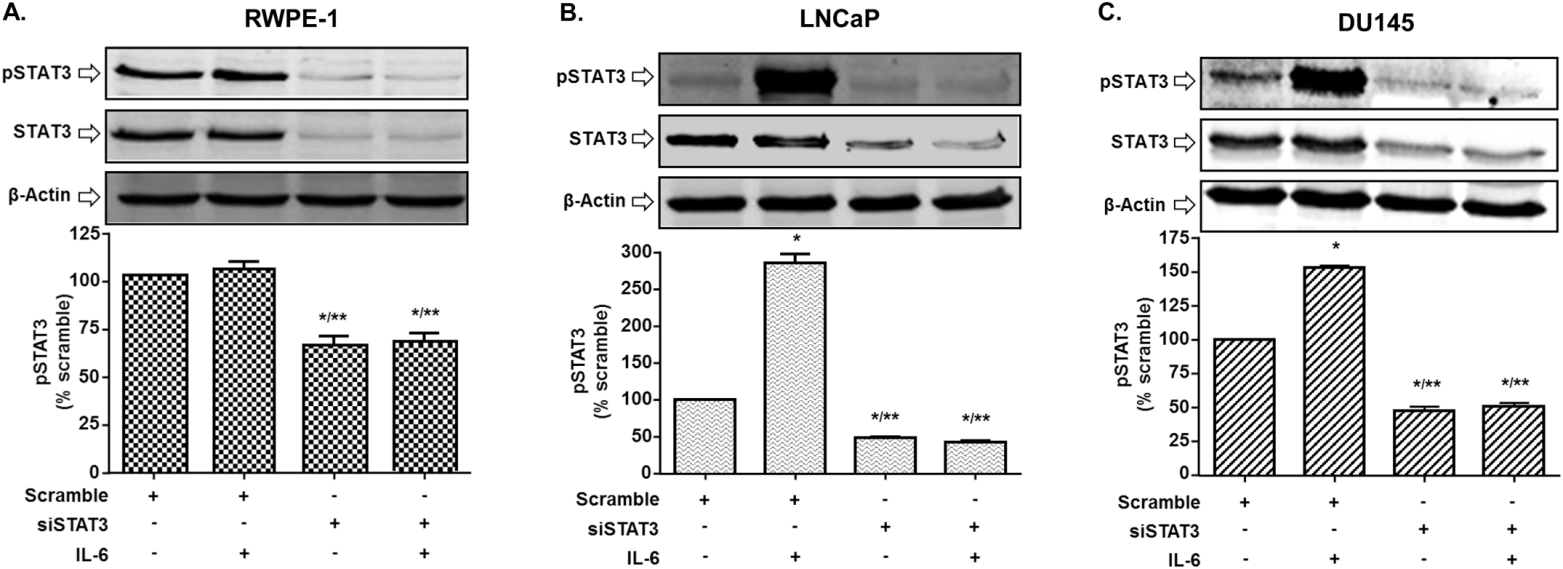
IL-6 induces STAT3 phosphorylation in prostate cancer cells. **(A)** RWPE-1 cells were transfected with either scramble or siSTAT3 (10 mM) for 24 h then subjected to IL-6 treatment (10 ng/ml) for 30 min, then cells lysate were used for Western blotting analysis. IL-6 showed no significant effect on STAT3 phosphorylation on this cell. **(B, C)** LNCaP and DU145 cells were transfected with a scrambled siRNA sequence or siSTAT3 (10 nM) for 24 h then treated with vehicle or IL-6 (10 ng/ml) for 5 min (DU145) or 30 min (LNCaP). Cells were then lysed and used for Western blotting analysis. Bar graph represents mean ± SEM of 3 independent experiments. Asterisks (*) (**) indicate statistically significantly difference (p < 0.05) from scramble or IL-6, respectively.

Since IL-6 signaling pathway includes STAT3, we performed Western blot studies to assess the status of p-STAT3 in the different prostate cancer cell lines following IL-6 treatments. Significant increases in p-STAT3 levels (normalized to STAT3) were observed following treatment with IL-6 (10 ng/ml) in DU145 and LNCaP cells. However, IL-6 treatment did not significantly alter STAT3 phosphorylation in RWPE-1 cells. Knockdown of STAT3 using siRNA, not only blunted the responses of IL-6 in DU145 and LNCaP cells but reduced basal pSTAT3 activities in all three cell lines. These data suggest that IL-6 activates STAT3 in DU145 and LNCaP cells but not in the normal RWPE-1 cell line (Fig 3A, 3B and 3C). These results suggest a positive correlation between p-STAT3 and IL-6 and suggest that pSTAT3 is involved in down-regulation of *PCAT29*.

**Fig 4:**
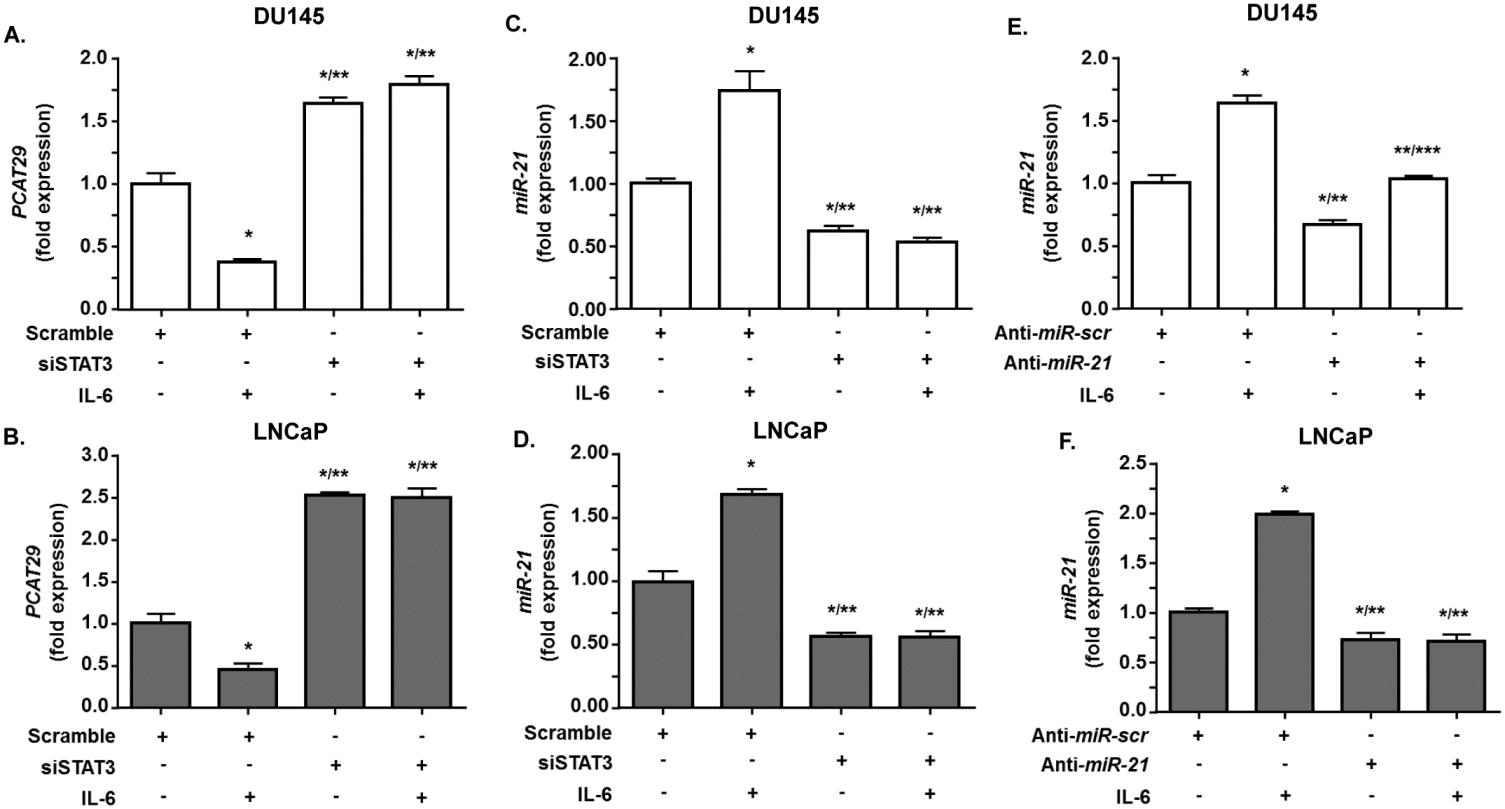
STAT3 knockdown increase the level of *PCAT29* expression. **(A, B)** DU145 and LNCaP cells were transfected with either scramble siRNA sequence or siSTAT3 (10 nM) for 24 h then treated with IL-6 (10 ng/ml) for another 24 h. Cells were then used to determine *PCAT29* level by real-time RT-PCR. IL-6 suppressed *PCAT29* expression in both DU145 and LNCaP cells which were pretreated with scrambled siRNA but not following transfection with siSTAT3. **(C, D)** DU145 and LNCaP cells were transfected with either scramble or siSTAT3 (10 nM) for 24 h, then treated with IL-6 (10 ng/ml) for 24 h, and used to determine *miR-21* levels by TaqMan^®^ real-time PCR. The levels of *miR-21* were increased by IL-6 in the cells treated with scrambled siRNAs but not after transfection with siSTAT3. In fact, siSTAT3 reduced both basal and IL-6 stimulated *miR-21* expression. **(E, F)** DU145 and LNCaP cells were transfected with a scrambled sequence (anti-*miR*-scr) or anti-*miR*-21 (30ng/ml) for 24 h and then treated with IL6 (10 ng/ml) for 24 h. The expression of *miR-21* was the determined by TaqMan^®^ real-time PCR. IL-6 increased *miR-21* in the group treated with the scrambled sequence but its effects were significantly reduced by anti-*miR-21* in DU145 cells and completely reversed in LNCaP cells. Data represent the mean ± SEM of at least 3 independent experiments. Asterisks (*), (**), and (***) indicate statistically significantly difference (p < 0.05) from scramble, from scramble +IL-6 and from anti-*miR-21*, respectively.

Additional studies were performed to confirm a role of STAT3 in regulation of *PCAT29* expression. Knockdown of STAT3 using siRNA resulted in increased levels of *PCAT29* in both DU145 and LNCaP cells. In addition, knockdown of STAT3 abolished the ability of IL-6 to decrease *PCAT29* (Fig 4A and 4B). The relative expression of *PCAT29* following STAT3 knockdown and treatment with vehicle or IL-6 were 1.64 ± 0.05-fold and 1.79 ± 0.07-fold over scramble siRNA, respectively, in DU145 cells. In LNCaP cells, the responses of IL-6 following STAT3 knockdown were 2.25 ± 0.03-fold and 2.47 ± 0.11-fold over scramble siRNA, respectively.

Our results also showed that IL-6 significantly increased *miR-21* levels. The relative increases in expression of *miR-21* were 1.84 ± 0.09 and 1.72 ± 0.04 over the control in DU145 and LNCaP cells, respectively. However, knockdown of STAT3 led to significantly decreased IL-6 stimulated *miR-21* levels in both DU145 and LNCaP cells as compare to control. The relative expression of *miR-21* following STAT3 knockdown were 0.61 ± 0.11 and 0.61 ± 0.03 in DU145 and LNCaP cells, respectively. In addition to relative expression of *miR-21* in STAT3 transfected groups treated with IL-6 were 0.54 ± 0.08 and 0.51 ± 0.13 in DU145 and LNCaP cells, respectively. (Fig 4C and 4D).

### *MiR-21* regulates *PCAT29* in prostate cancer cells

**Fig 5:**
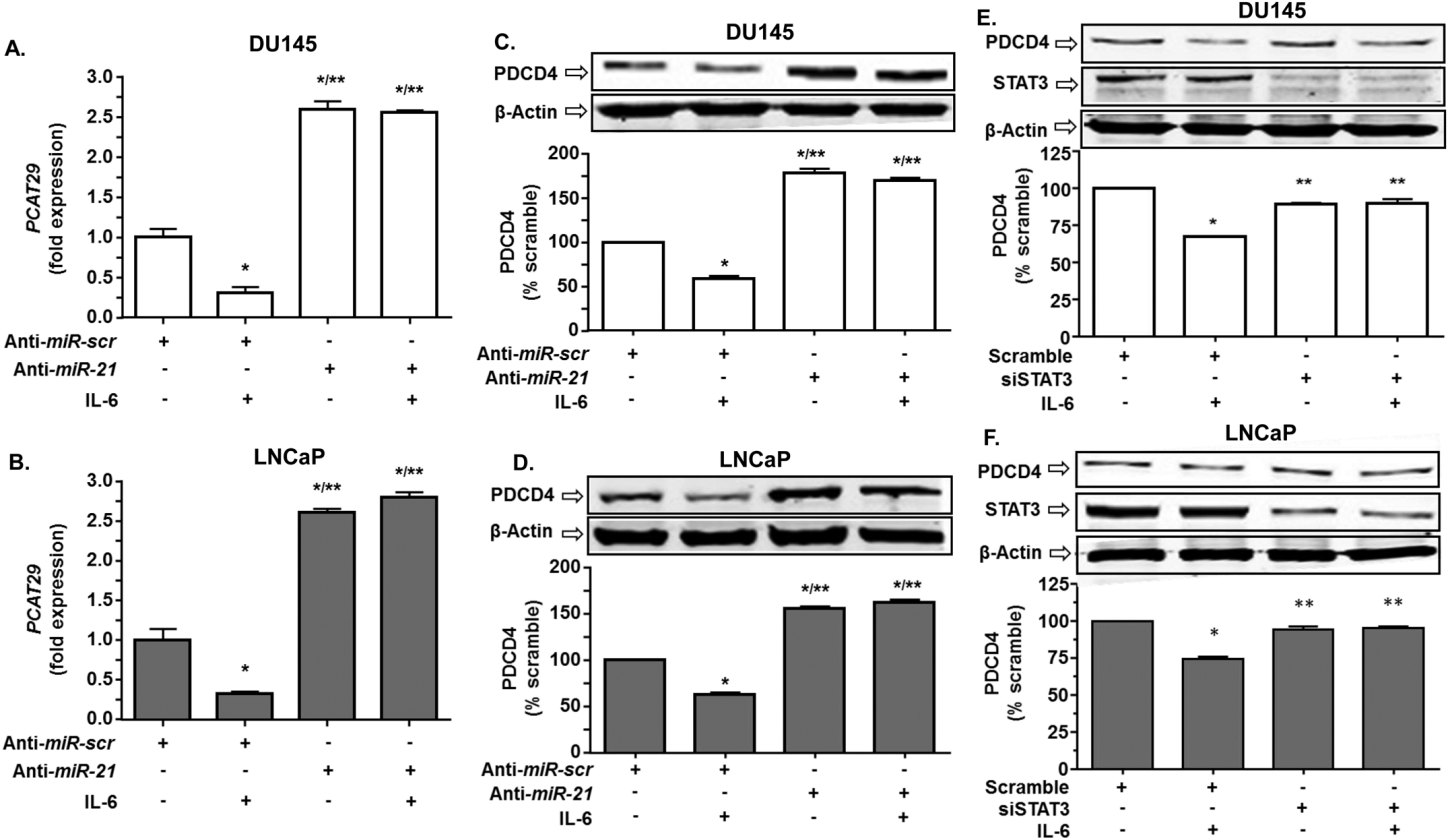
Knockdown of *miR-21* increases the levels of *PCAT29* and PDCD4. **(A, B)** DU145 and LNCaP cells were transfected with a scrambled oligo sequence (anti-*miR-scr*) or anti-*miR-21* (30 nM) for 24 h. Cells were then treated with vehicle or IL6 (10 ng/ml) for 24 h and the expression of *PCAT29* were determined by real-time RT-PCR. IL-6 significantly decreased the expression of *PCAT29* in the anti-*miR-scr*. Knockdown of *miR-21* enhanced the expression of *PCAT29* and abolished the effect of IL-6 in both cell lines. **(C, D**) DU145 and LNCaP cells were transfected with anti-*miR-21* (30 nM) for 24 h and then treated with IL-6 (10 ng/ml) for another 24 h. Cell lysates were prepared and used for Western blotting analysis. IL-6 significantly decreased the expression of PDCD4. Knockdown of *miR-21* increased basal PDCD4 levels and abolished the reduction of this protein by IL-6. **(E, F)** DU145 and LNCaP cells were transfected with siRNA against STAT3 (10 nM) for 24 h. Cells were then treated with IL-6 (10 ng/ml) for 24 h and lysates were used for Western blotting analysis. IL-6 reduced PDCD4 levels, but this effect was abolished by siSTAT3. Data are presented as the mean ± SEM of at least 3 independent experiments. Asterisks (*) and (**) indicate statistically significantly difference (p < 0.05) from scramble and from scramble +IL-6 treatment groups, respectively.

Previous studies have shown that *miR-21* is a relevant target of STAT3, which mediates growth factor receptors regulation of cell proliferation. Additional studies show that lncRNAs can be regulated by microRNA [3]. We first investigated the efficiency of *miR-21* knocking down using *anti-miR-21* oligonucleotides. This resulted in significantly reduced *miR-21* expression in both DU145 and LNCaP by 33.4 ± 3.8% and 27.3 ± 3.5%, respectively. IL-6 treatment increased *miR-21* levels by 1.64 ± 0.04 and 1.98 ± 0.07-fold over vehicle control in DU145 and LNCaP cells, respectively. However, the responses of these cells to IL-6 were reduced (in DU145) or completely abrogated (in LNCaP) following partial knockdown of *miR-21* (Fig 4E and 4F). Measurement of *PCAT29* expression in these cells showed that IL-6 suppressed *PCAT29* expression in DU145 and LNCaP cells and the relative expression were reduced to 0.30 ± 0.01-fold and 0.32 ± 0.03-fold, respectively. Partial knockdown of *miR-21* resulted in significant increases in the basal levels of *PCAT29* and blunted its reduction by IL-6 in DU145 and LNCaP cells. The relative expressions of *PCAT29* were 2.59 ± 0.03-fold and 2.6 ± 0.05-fold over control following partial knockdown of *miR-21* and treatment with IL-6 in DU145 and LNCaP cells, respectively. There were no significant differences in *PCAT29* levels in the *miR-21* knockdown groups treated with either vehicle or IL-6 (Fig 5A and 5B). These results provide good evidence that *miR-21* is an important regulator of *PCAT29* expression. The almost complete abolition of the IL-6 response following partial knockdown of *miR-21* suggests that *PCAT29* expression is very sensitive to changes in *miR-21* levels in prostate cancer cells.

We next examined the levels of PDCD4, a downstream target negatively regulated by miR-21. Our results also show a negative correlation between *miR-21* and PDCD4. IL-6 treatment reduced PDCD4, as anticipated due to the ability of this cytokine to increase *miR-21* levels. Knockdown of *miR-21* resulted in a significant increase of PDCD4 protein levels in both DU145 and LNCaP cells. IL-6 treatment showed decrease in PDCD4 in both DU145 and LNCaP cells (Fig 5C and 5D). In addition, partial knockdown of STAT3 resulted in increased levels of PDCD4 and abrogated the effect of IL-6 (Fig 5E and 5F). Overall, these data support the above finding that *miR-21* could regulate the expression of *PCAT29* in prostate cancer cells.

### Resveratrol blocks IL-6 effects on prostate cancer cells

**Fig 6:**
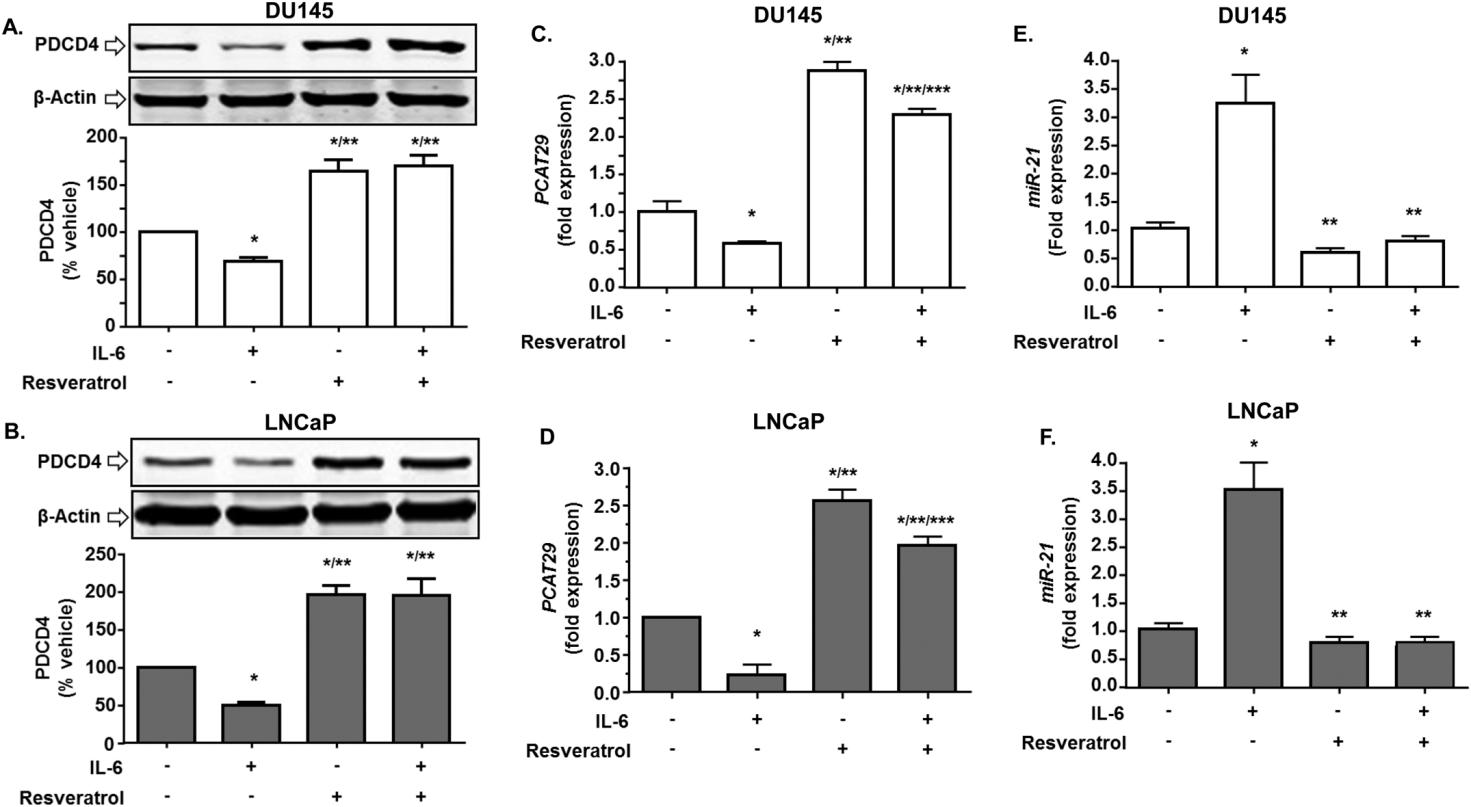
Resveratrol blocks IL-6 suppression of PDCD4 and *PCAT29* and its induction of *miR-21*. **(A, B)** Western blotting analysis of DU145 and LNCaP cells showed that IL-6 reduced the levels of PDCD4. Resveratrol (25 μM) treatment for 24 h blocked this reduction and increased the levels of PDCD4 above that of control. This effect of resveratrol persisted even in the presence of IL-6. **(C, D)** Resveratrol also increased *PCAT29* by greater than 2-fold and this effect was partly reduced by concurrent IL-6 treatment. **(E, F)** Resveratrol restored IL-6 stimulated expression of *miR-21* in LNCaP and DU145 cells. Data are presented as the mean ± SEM of at least 3 independent experiments. Asterisks (*), (**), and (***) indicate statistically significantly difference (p < 0.05) from vehicle, from vehicle+IL-6 and from resveratrol, respectively.

We have previously shown that the anti-tumor actions of resveratrol are mediated, in part, by inhibiting the *miR-21* signaling pathway in prostate cancer [34]. Since IL-6 regulation of *PCAT29* appears dependent on *miR-21*, we tested the effect of resveratrol on *PCAT29* expression. One important gene which is negatively regulated by *miR-21* is the tumor suppressor programmed cell death protein 4 (PDCD4) [16] which is induced by resveratrol [34]. As shown above, IL-6 decreased the levels of PDCD4 following 24 h treatment in DU145 and LNCaP cells, while knockdown of *miR-21* increased PDCD4 levels in both vehicle- and IL-6 treated cells and abolish IL-6 effect (Fig 5C and 5D). Additional studies show that the reductions in PDCD4 produced by IL-6 treatment was reversed by resveratrol (Fig 6A and 6B). In fact, resveratrol significantly increased PDCD4 levels above control levels. In addition, in presence of resveratrol, IL-6 was unable to decrease *PCAT29* expression in both DU145 and LNCaP cells. The fold expression of *PCAT29* for the resveratrol-pretreated groups in absence and presence of IL-6 were 2.87 ± 0.12 and 2.2 ± 0.08, respectively for DU145 cells. In LNCaP cells the fold expression of *PCAT29* were 2.56 ± 0.15 and 1.9 ± 0.11 in vehicle and IL-6 treatment groups, respectively (Fig 6C and 6D). Furthermore, we show that while IL-6 increased *miR-21* in these cells, the addition of resveratrol (25μM) for 24 h led to significant reductions in both basal and IL-6 induced *miR-21* levels (Fig 6E and 6F). Thus, resveratrol induction of *PCAT29* expression involves suppression of IL-6/STAT3/*miR-21* signaling.

### *PCAT29* regulates oncogenic phenotypes *in vitro*

**Fig 7:**
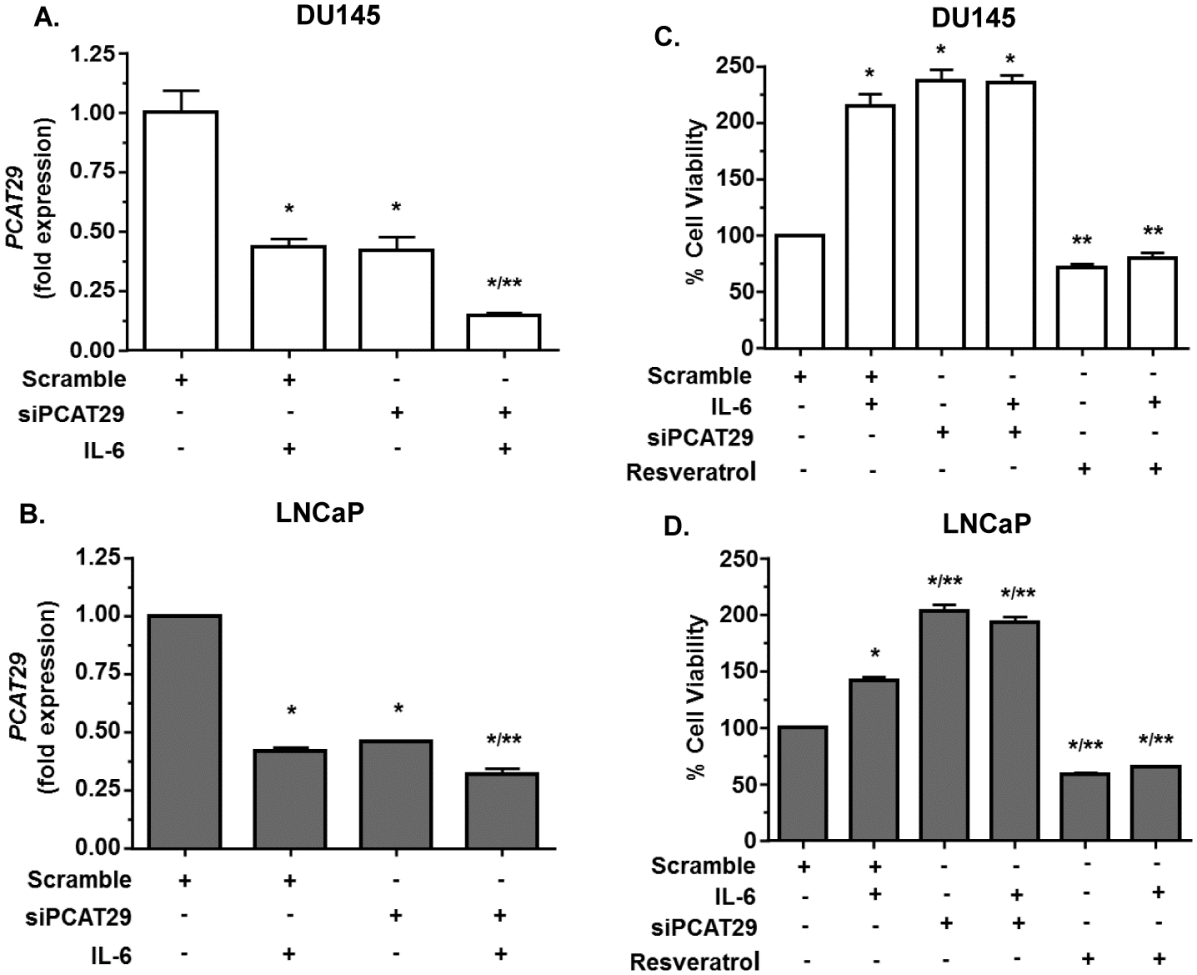
*PCAT29* tonically suppressed prostate cancer cell proliferation. **(A, B)** DU145 and LNCaP cells were transfected with scrambled siRNA or siRNA against *PCAT29* (si*PCAT29*, 30 nM) for 24 h, followed by treatment with IL6 (10 ng/ml) for 24 h. Cells were then used to determine *PCAT29* levels. **(C, D)** DU145 and LNCaP cells were transfected with scrambled siRNA or si*PCAT29* (30 ng/ml) for 24 h, followed by treatment with IL-6 for 24 h and cells proliferation was determined by MTS assay. IL-6 treatment significantly increased cells proliferation, which was mimicked by si*PCAT29*. Knockdown of *PCAT29* did not increase cell proliferation above that observed with IL-6 alone in DU145 but not in LNCaP cells. Resveratrol treatment significantly reduced cell proliferation, even in presence of IL-6. Data are presented as the mean ± SEM of at least 3 independent experiments. Asterisks (*) and (**) indicate statistically significantly difference (p < 0.05) from scramble and from scramble + IL-6, respectively.

Previous studies showed *PCAT29* is an important gene for inhibiting prostate cancer [32]. Since *PCAT29* is negatively regulated by IL-6 and positively regulated by resveratrol, we decided to investigate the functional role of *PCAT29*. First siRNA was designed to knockdown the expression of *PCAT29* in prostate cancer cells. Using siRNA, we shown a significant reduction in *PCAT29* level in both DU145 and LNCaP cells, by 60 ± 3% and 54 ± 2%, respectively. IL-6 treatment decreased *PCAT29* levels by 26 ± 1% and 41 ± 1% in DU145 and LNCaP cells, respectively (Fig 7A and 7B). Knockdown of *PCAT29* led to increased proliferation in both DU145 and LNCaP cells compared to increased cell proliferation obtained with IL-6 in normal cells. Resveratrol abolished both basal and IL-6 dependent cell proliferation in both DU145 and LNCaP cells (Fig 7C and 7D).

### Human prostate cancer specimen demonstrate increase in pSTAT3

**Fig 8:**
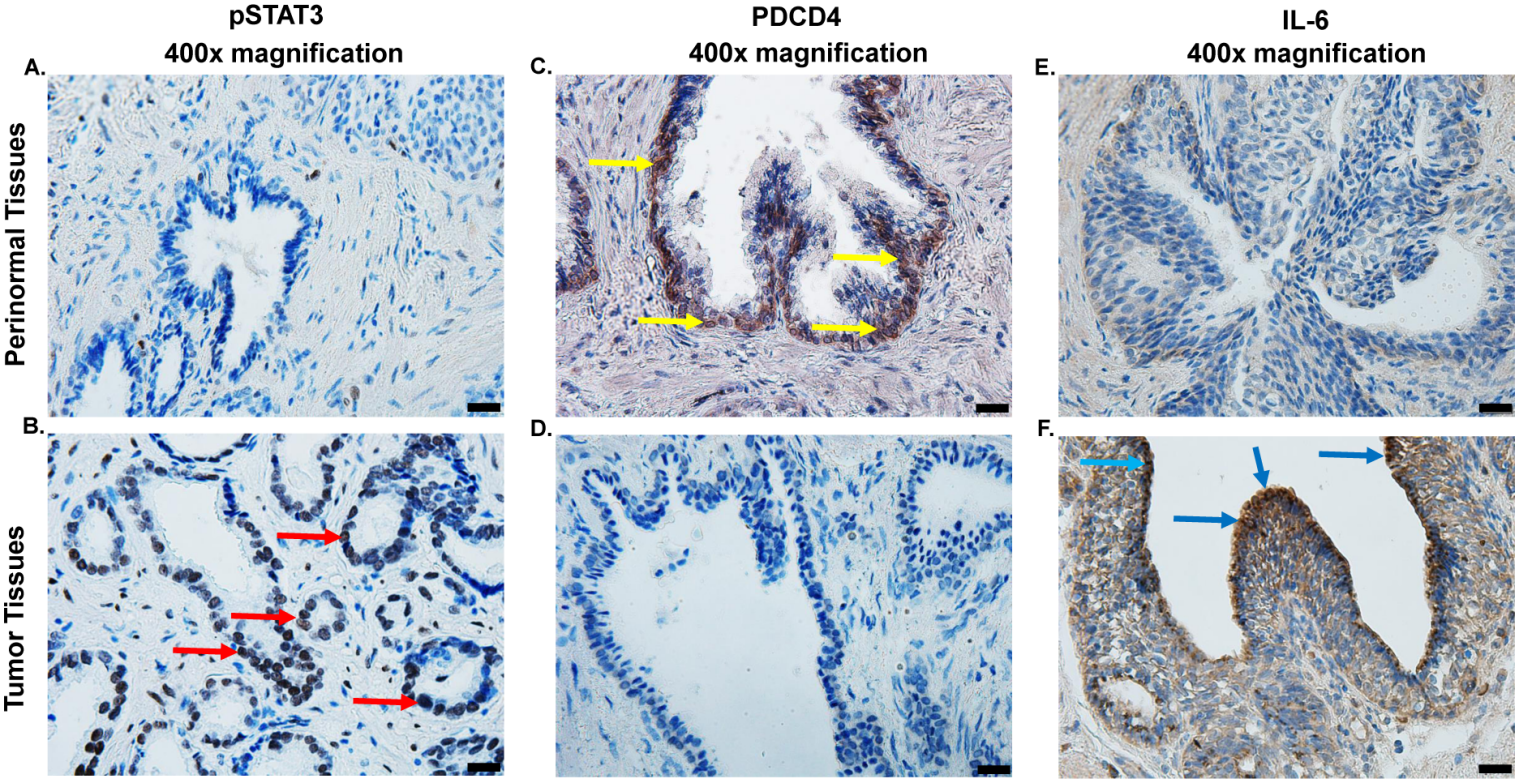
Levels of pSTAT3, PDCD4 and IL-6 in human prostate specimens. **(A, B)** Immunohistochemistry studies were performed to determine the levels of pSTAT3 in formalin-fixed paraffin embedded prostate tumor specimens, as compared to adjacent normal prostate specimen. Diaminobenzidine tetrahydrochloride (DAB) staining revealed high expression of pSTAT3 **(B)** as dark-brown labeling in epithelial cells of tumor samples (marked by red arrows) while low expression was observed in paired perinormal prostate tissues **(A)**. In contrast, high expression of PDCD4 was observed in perinormal prostate tissues (as evidenced by dark-brown labeling, indicated by yellow arrows) **(C)**, as compared to prostate tumors **(D)**. Immunolabling of prostate tumor revealed high expression of IL-6 **(F)**, compared to perinormal prostate specimens. Representative images show immunohistochemical studies performed in four different specimens obtained from three different patients. Pictures magnification is 400x. Scale bar is 20 μm.

STAT3 acts as an oncogenic tumor marker in prostate and colon cancer tissues, where it mediates hyperplasia and neoplastic transformation [37]. The role of STAT3 as a downstream conduit of IL-6 signaling and as a regulator of *miR-21* prompted us to determine whether pSTAT3 levels was inversely correlated with PDCD4 in human prostate specimens. Immunohistochemical comparisons were made between prostate cancer and its paired perinormal tissue using paraffin embedded sections. We observed a higher degree of pSTAT3 staining in prostate cancer than those in perinormal tissues (Fig 8A). Immunolabeling for PDCD4 showed high expression of this protein in normal specimens, compared to lower levels of this protein in prostate cancer (Fig 8B). In addition, we observed localization of PDCD4 mainly in nuclei of epithelial cells in normal but not in these cells in prostate cancer specimens. In addition, immunohistochemical comparison between normal and prostate cancer using paraffin embedded sections show a higher degree of IL-6 staining in prostate cancer than those in perinormal tissue sections (Fig 8C). Overall, data from human prostate specimens indicate a reciprocal relationship between the levels of pSTAT3 and IL-6 versus those of PDCD4.

## Discussion

There is relationship between inflammation and cancer development, as pro-inflammatory cytokines participate in the progression of cancer cells to a more aggressive form [38]. In particular, the IL-6/STAT3 signaling pathway appears to be crucial for the progression of prostate cancer. High levels of IL-6 expression is detected in patients with prostate cancer [16,37] which could activate IL-6 receptor, leading to increased STAT3 activation [39]. The levels of STAT3 have also been found to be elevated in many cancers where they stimulate cell proliferation (via cyclin D1) and induction of anti-apoptotic proteins (such as Bcl2) [40]. Our findings show that IL-6 is a negative regulator of *PCAT29* expression which is mediated by activation of STAT3. Activated STAT3 promoted down-regulation of *PCAT29* expression in both androgen dependent and independent prostate cancer cells by induction of *miR-21*. Moreover, we showed reduced expression of *LncPCAT29* in Grade 4 prostate tumor samples compared to prostate samples from normal. These studies identify *PCAT29* as a novel target of *miR-21*, which could contribute to its pro-oncogenic properties. The current study shows another target of IL-6, namely *LncPCAT29*, whose down-regulation requires an interaction between STAT3 and *miR-21*. Down-regulation of *PCAT29* was associated with increased STAT3 and decreased levels of PDCD4 (a downstream target of *miR-21*) in Grade 4 prostate cancer. Previous studies have shown IL-6 can regulate the expression of *LncTCF7* in hepatocellular carcinoma via a STAT3 pathway [41]. Similarly, the IL-6/STAT3 pathway is implicated in the induction of *HOTAIR* LncRNA in human bronchial epithelial cells [42]. The expression of *HOTAIR* LncRNA was found to be regulated by a direct interaction of *miR-34a* with this gene [43]. *MiR-21* regulates prostate cancer progression by down-regulating tumor suppressor genes, such as PTEN and PDCD4 [44]. This is the first study to show that *PCAT29* is also a relevant target of *miR-21* in prostate cancer cells, as inhibition of *miR-21* expression leads to stimulation of *PCAT29*.

Resveratrol is generally regarded as a chemo-preventive agent for various diseases. This property is derived from the interaction of resveratrol with multiple targets and many molecular pathways [34,45]. The structure of resveratrol is similar to androgen and estrogen, suggesting that its might produce its beneficial actions via these hormone receptors [46]. We have previously shown that resveratrol reduces prostate cancer growth and metastasis by inhibiting the AKT/*miR-21* pathway [34]. In the current study, we show that targeting *miR-21* could also contribute to the induction of *PCAT29* by resveratrol. As such, resveratrol was shown to induce *miR-21* targets such as PDCD4 in both LNCaP and DU145. These findings suggest that *miR-21* could provide tonic suppression of *PCAT29* expression in prostate cancer cells which could be reversed by resveratrol.

*MiR-21* is an important cancer risk factor whose expression is induced by IL-6 [47,48]. Overexpression of *miR-21* is observed in many types of cancers, including, pancreas, lung, breast and prostate cancer [49]. This microRNA possesses oncogenic properties as it induces many proteins associated with cell proliferation [50,51]. Previous studies have shown that STAT3 binds directly to the *miR-21* promoter to induce its expression [52]. Our qPCR resulted showed that resveratrol down regulate *miR-21* expression in prostate cancers cells, presumably by suppressing STAT3. Thus, by interfering with the STAT3/*miR-21* pathway, resveratrol could block IL-6 mediated down-regulation of *PCAT29* and reduce tumorigenesis.

The overall impact of this study is significant as is provide strong evidence for the suppression of *PCAT29* expression by the IL-6 signaling pathway. This study identifies STAT3 and *miR-21* as important players in the regulation of *PCAT29*. Furthermore, it provides a clinically relevant drug, resveratrol, which could abrogate IL-6 signaling to boost the expression of *PCAT29* and facilitate its anti-tumor action.

## Supporting information

S1 Fig Original, uncropped and unadjusted images of blots mentioned in Figs 3A, 3B, 3C, 5C, 5D, 5E, 5F, 6A and 6B, respectively. (PDF)
