# Supplementary material for: Tonic suppression of PCAT29 by the IL-6 signaling pathway in prostate cancer: Reversal by resveratrol
Source: PLoS One. 2017 May 3;12(5):e0177198. doi: 10.1371/journal.pone.0177198 (PMC5415196; doi:10.1371/journal.pone.0177198)

Uncut and unedited image of the blot used in Figure 3A.

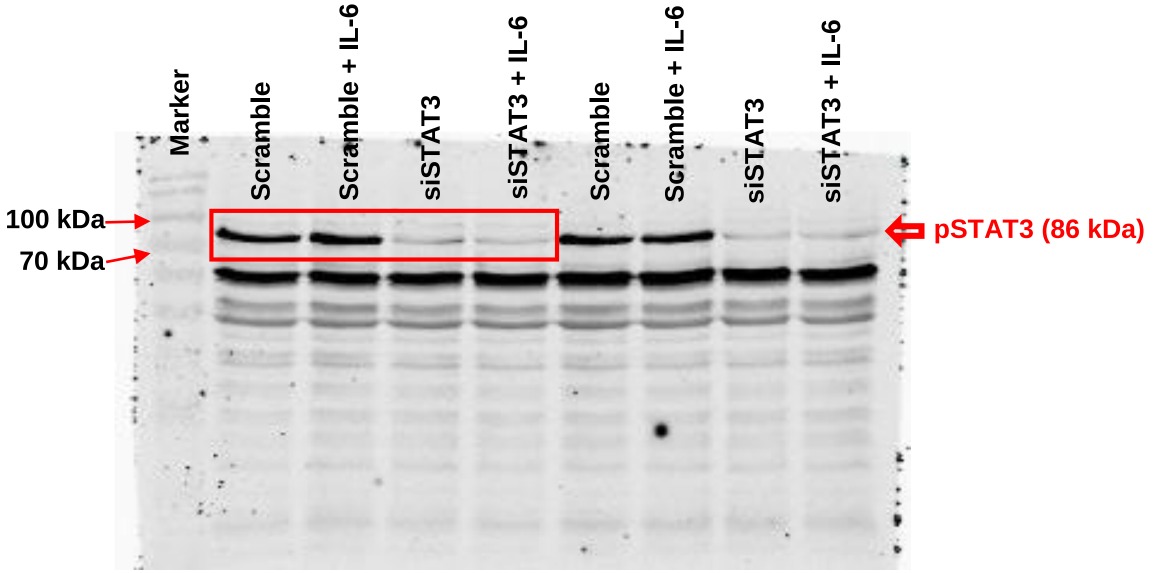

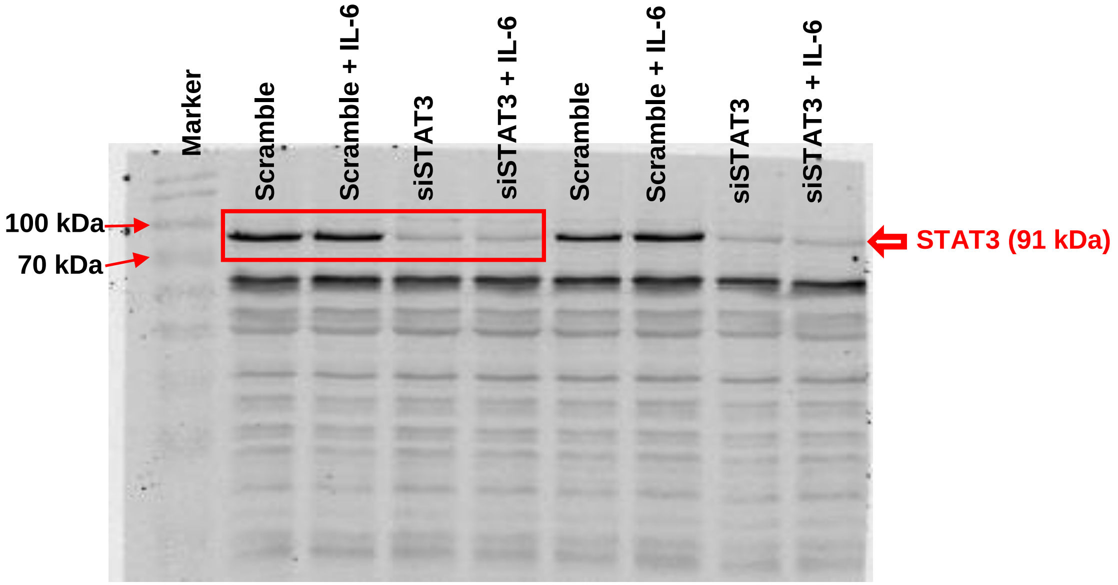

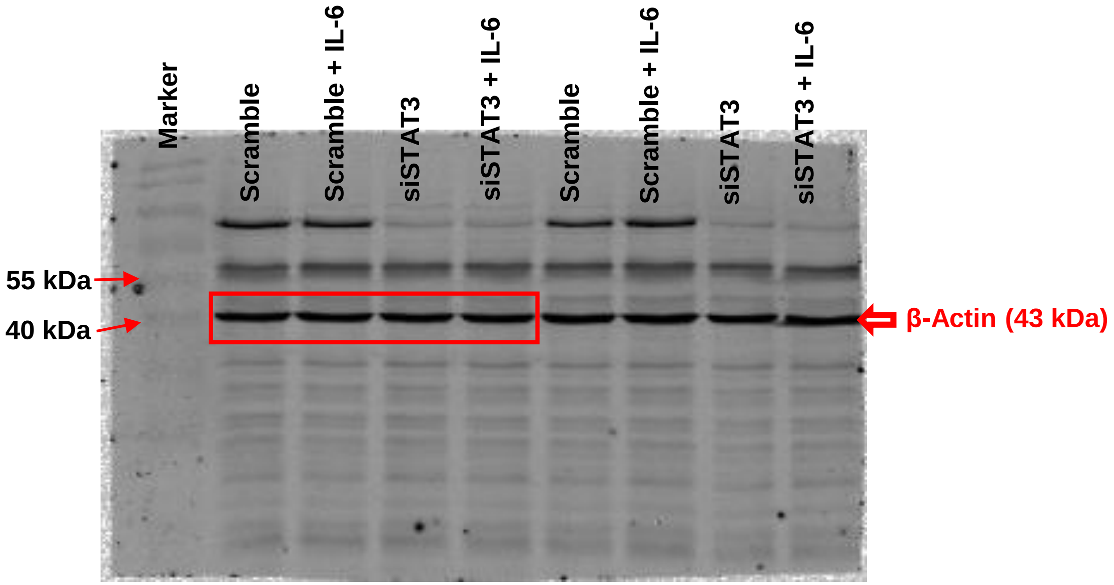

Uncut and unedited image of the blot used in Figure 3B

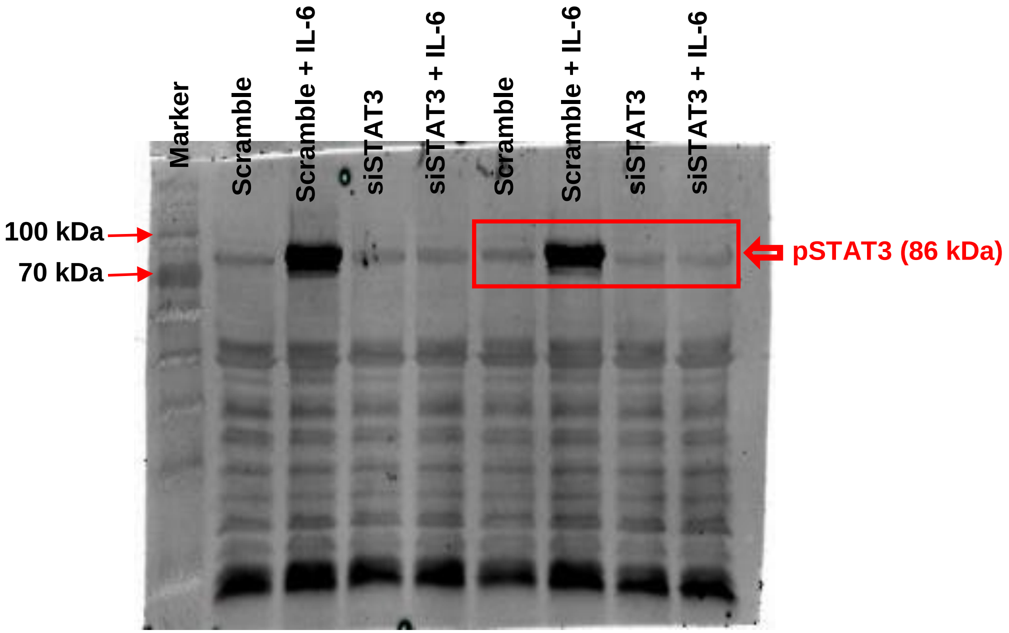

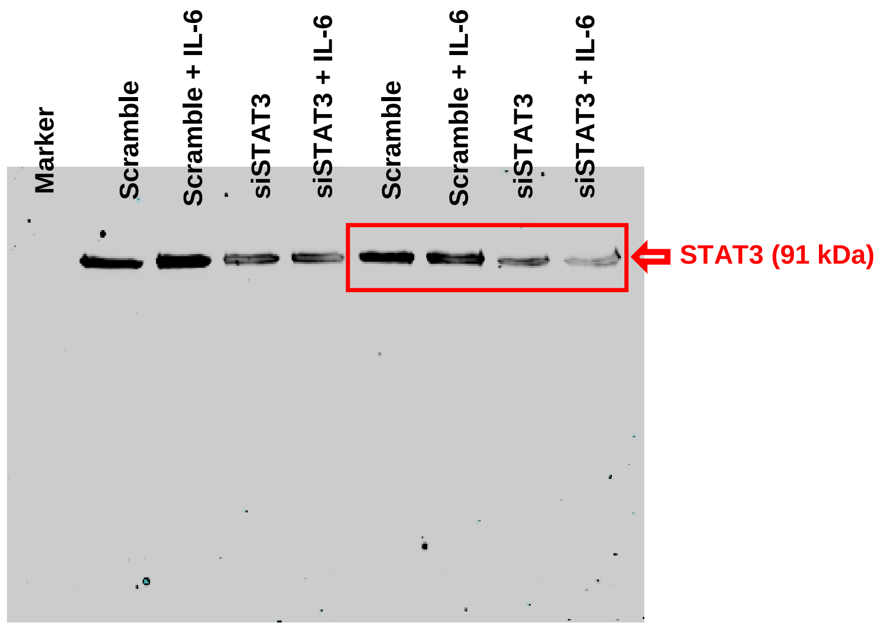

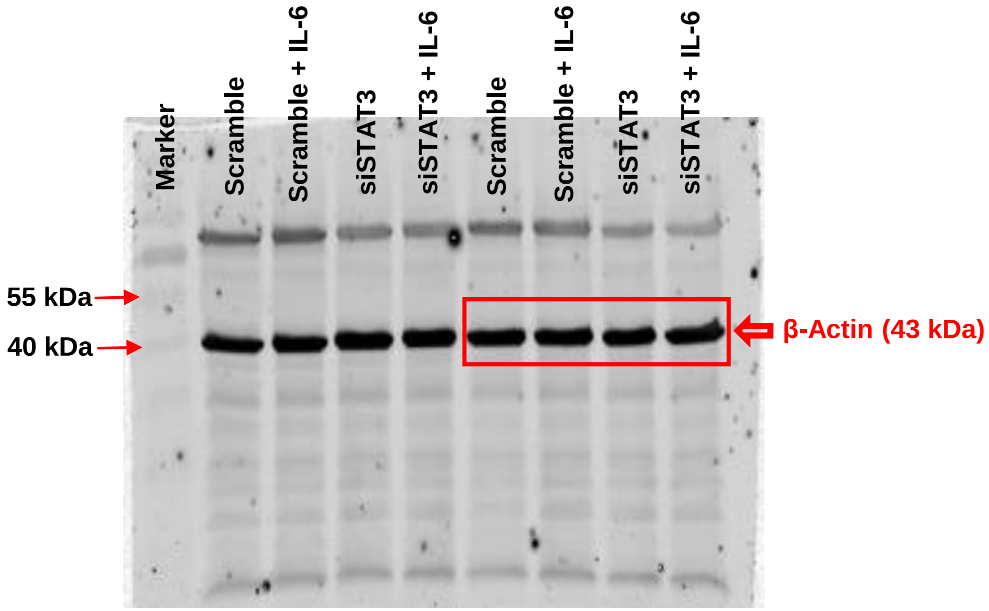

Uncut and unedited image of the blot used in Figure 3C

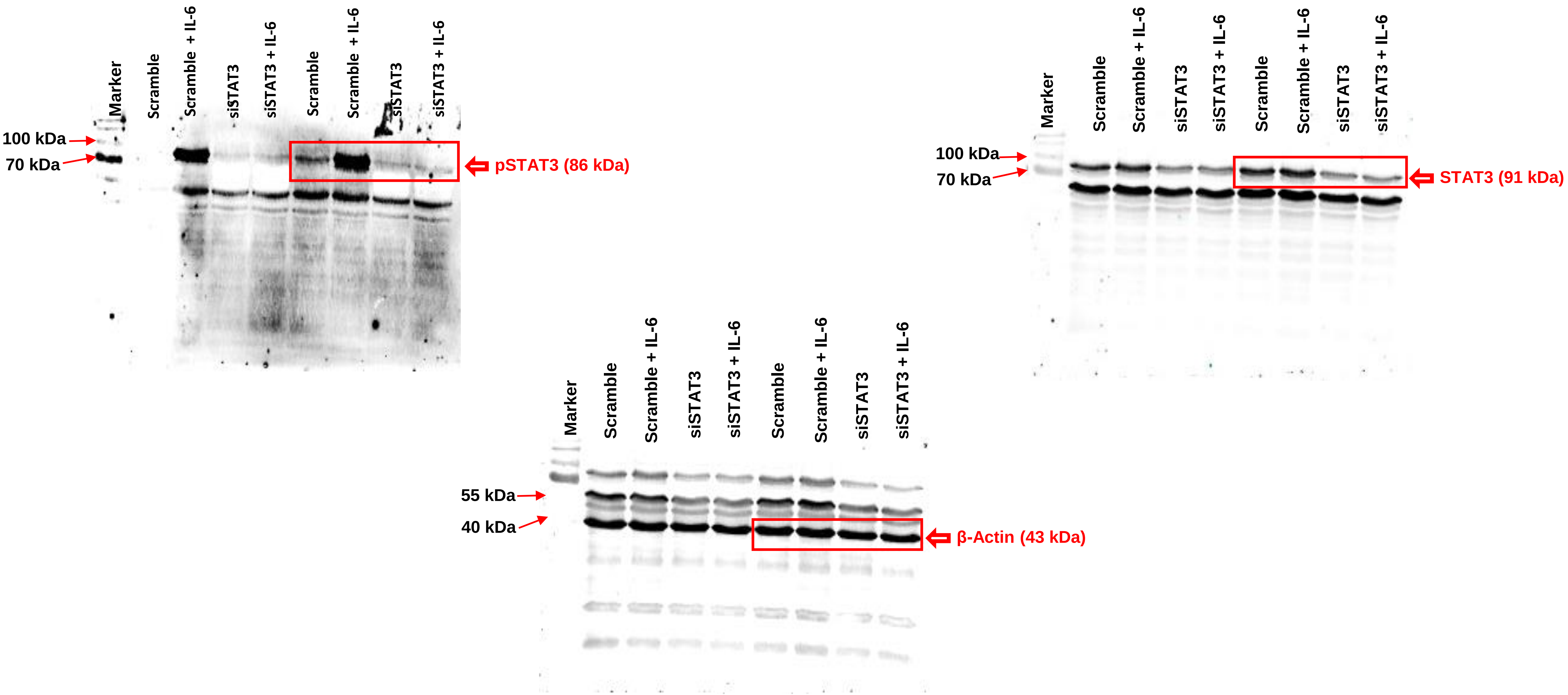

Uncut and unedited image of the blot used in Figure 5C

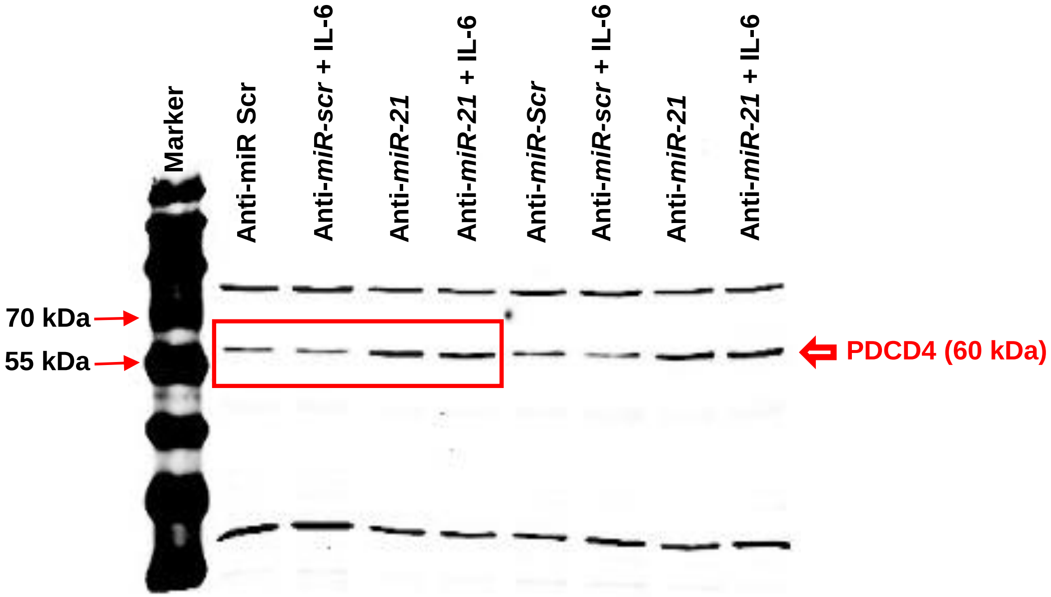

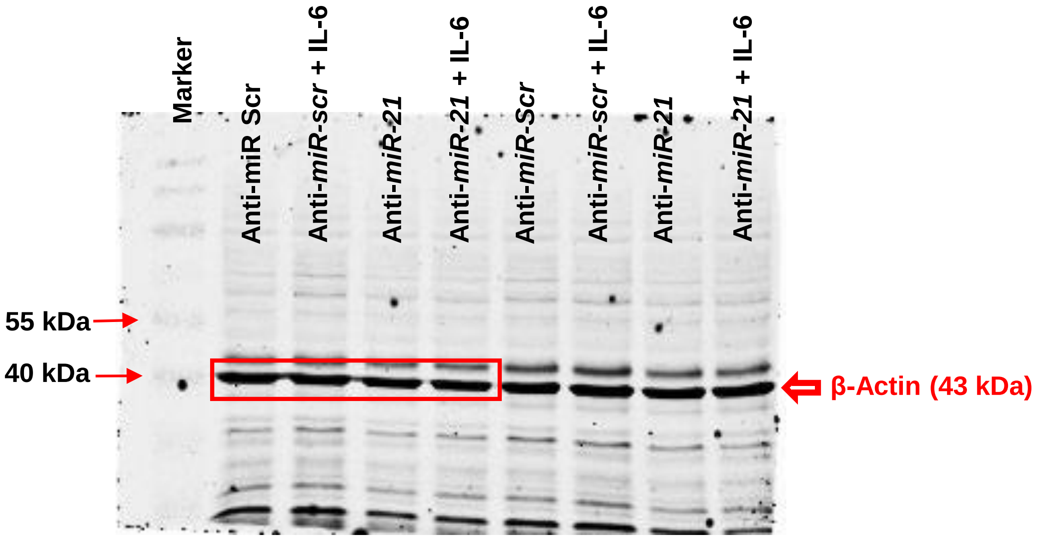

Uncut and unedited image of the blot used in Figure 5D

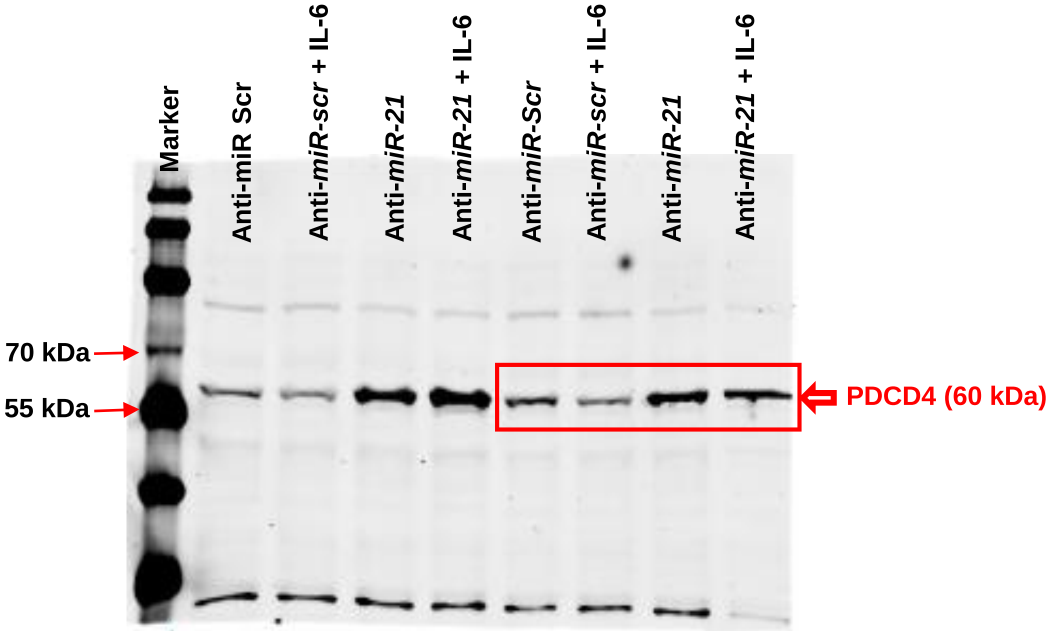

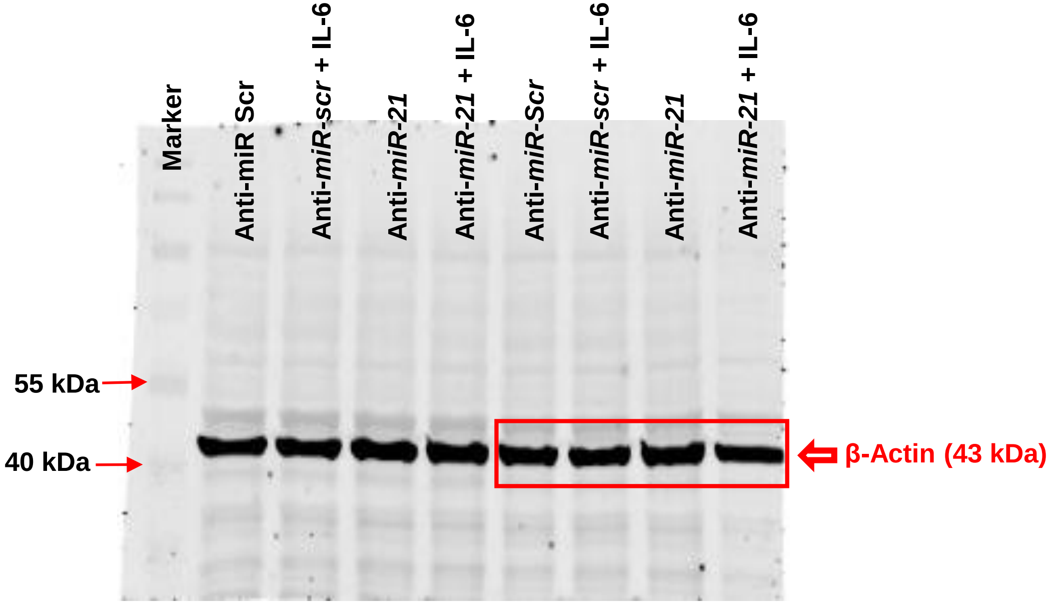

Uncut and unedited image of the blot used in Figure 5E

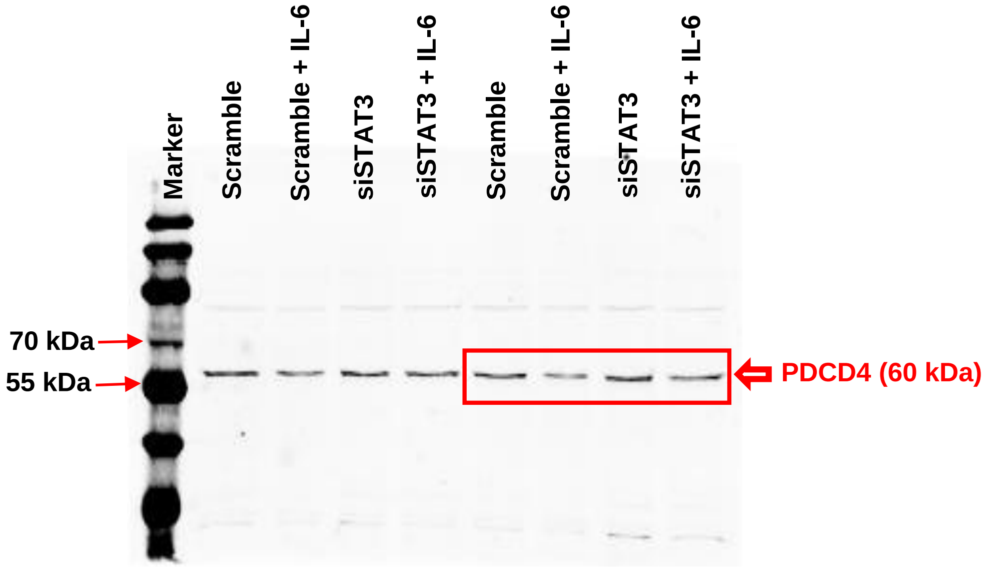

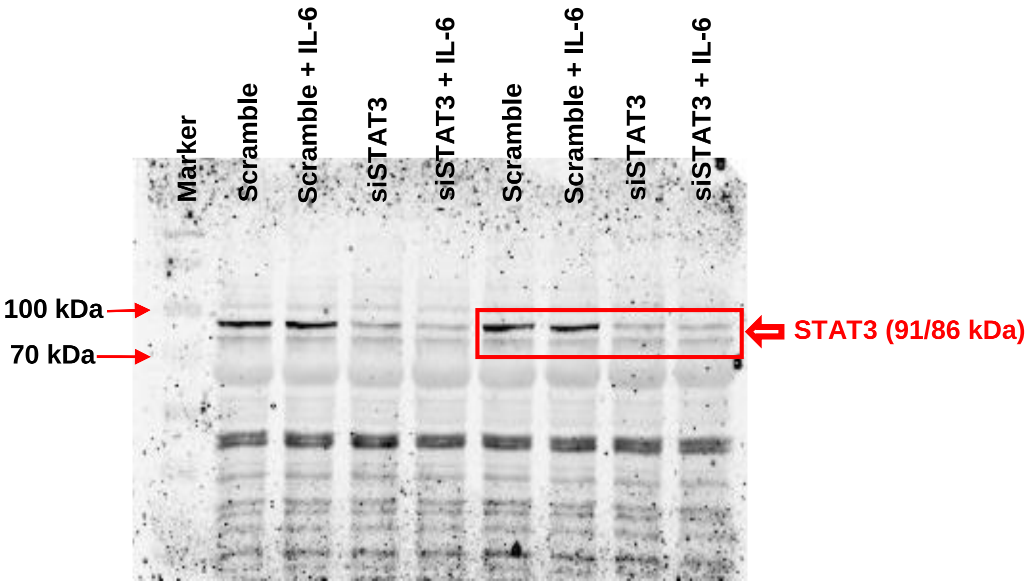

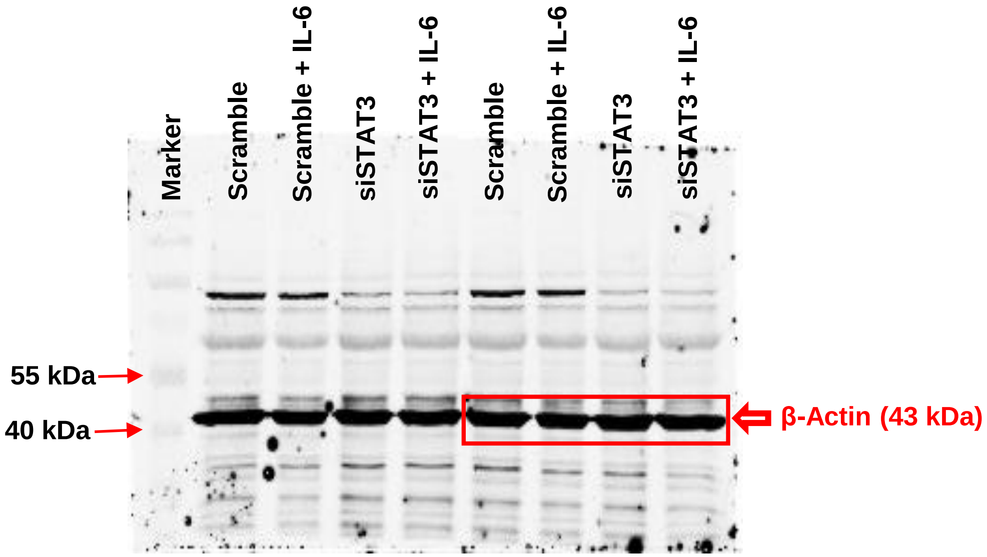

Uncut and unedited image of the blot used in Figure 5F

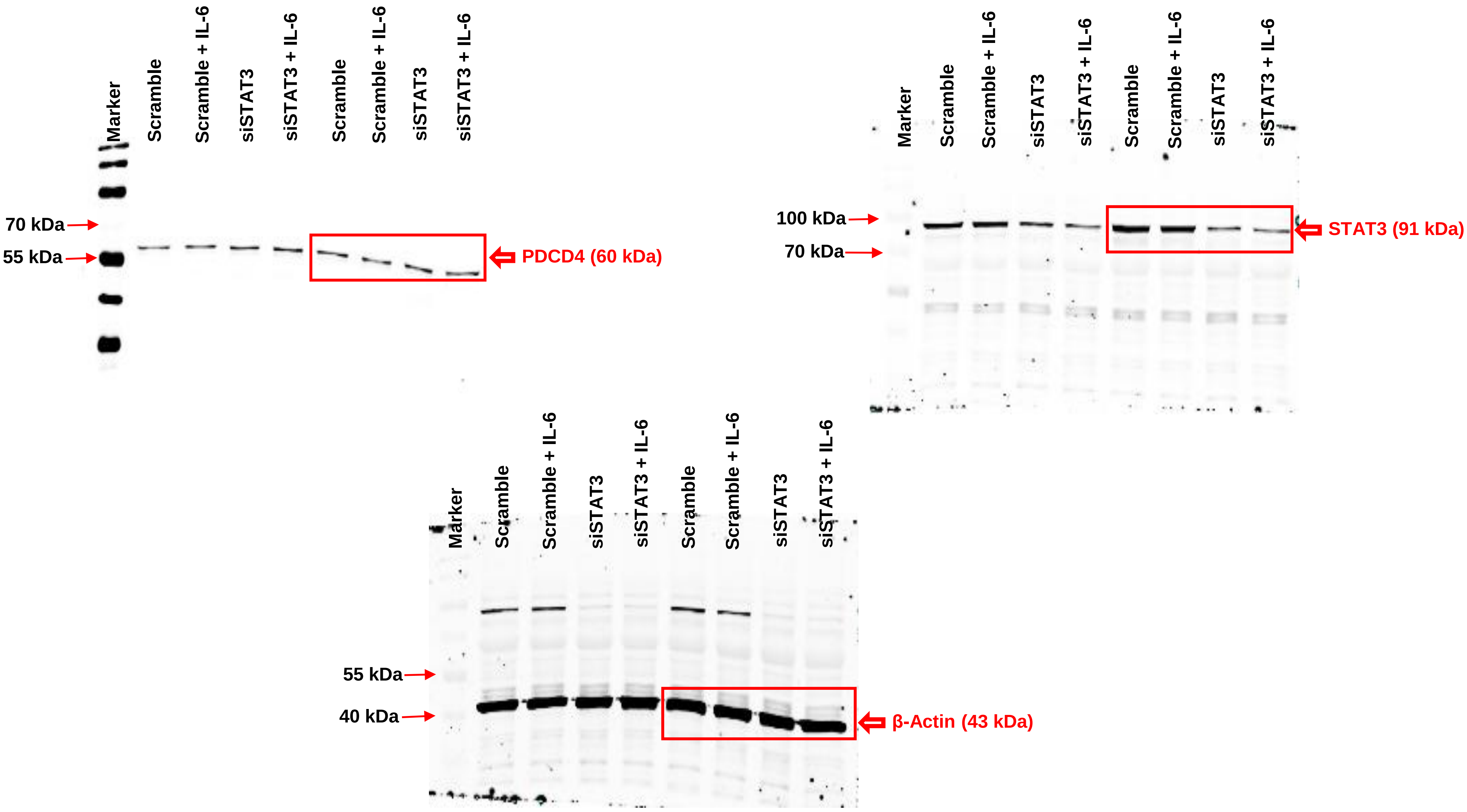

Uncut and unedited image of the blot used in Figure 6A

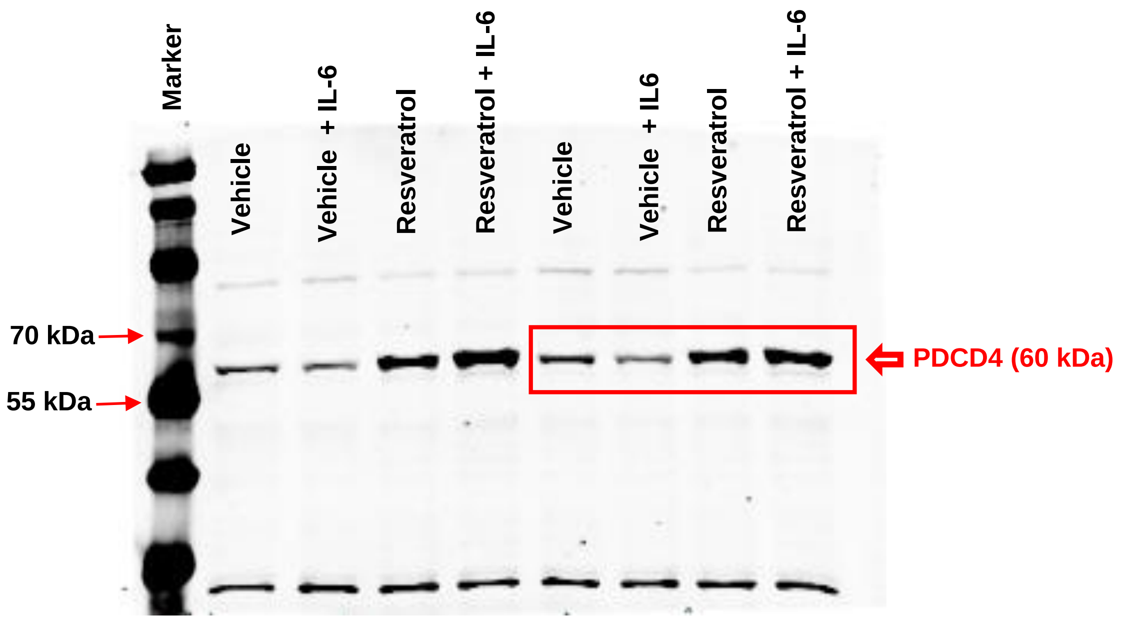

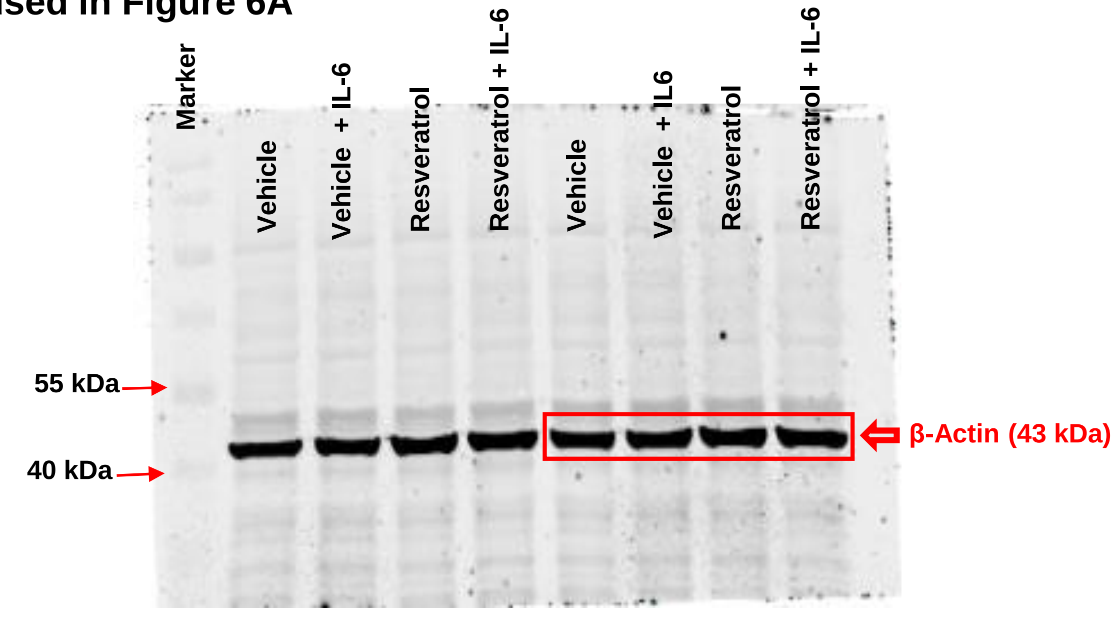

Uncut and unedited image of the blot used in Figure 6B

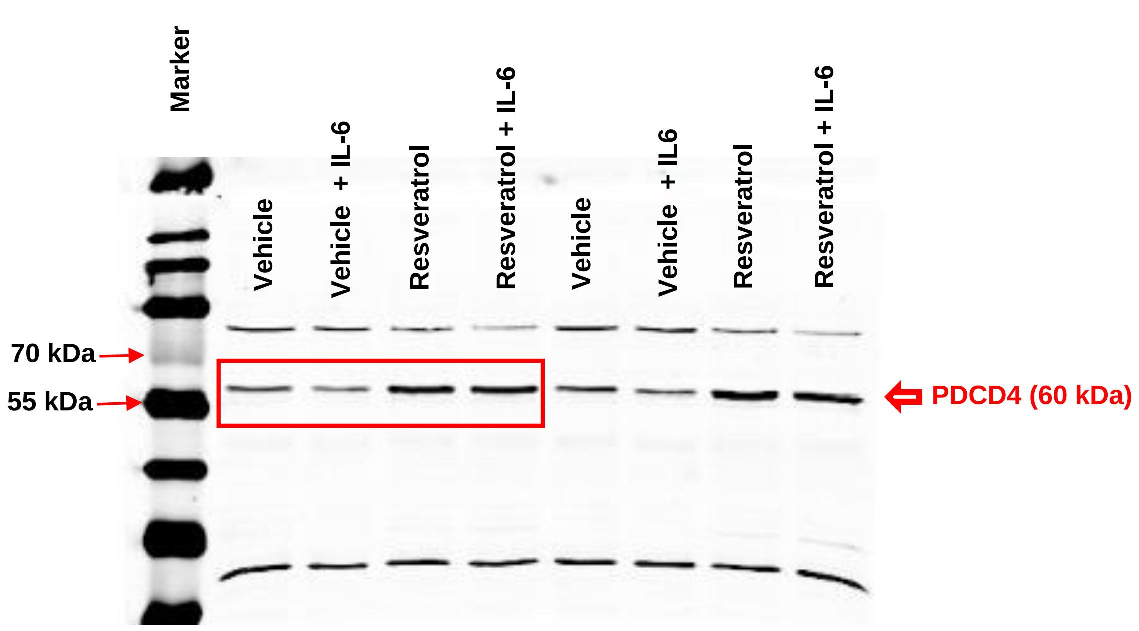

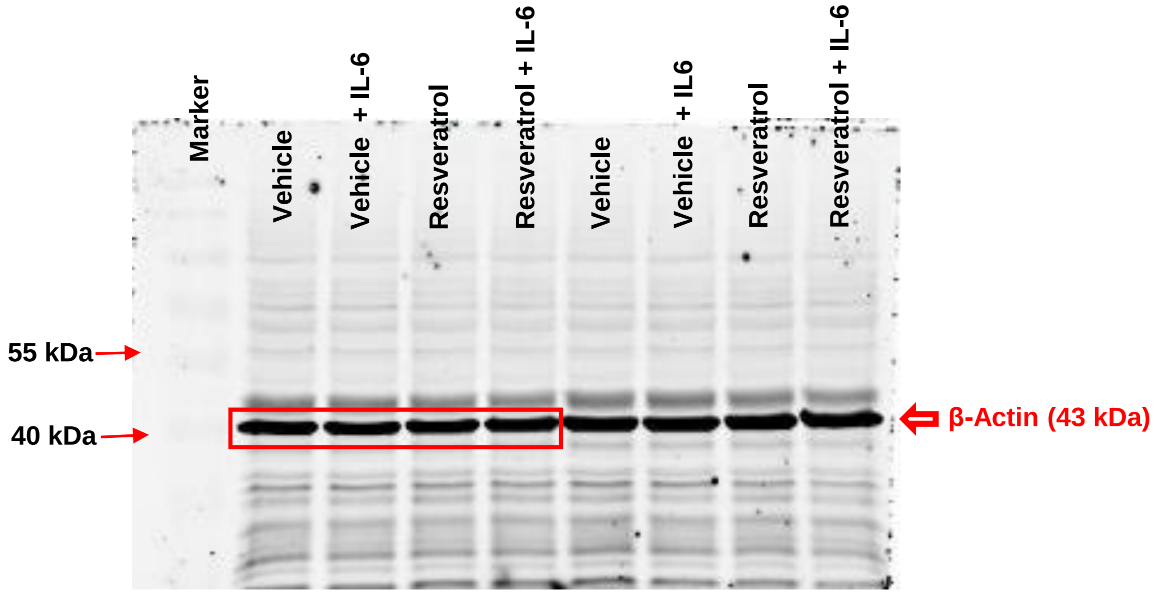

Supplement: S1 Fig — (PDF) [file pone.0177198.s001.pdf]
